# Increased Mesenchymal Stem Cell Functionalization in Three-Dimensional Manufacturing Settings for Enhanced Therapeutic Applications

**DOI:** 10.3389/fbioe.2021.621748

**Published:** 2021-02-12

**Authors:** Dimitrios Kouroupis, Diego Correa

**Affiliations:** ^1^Department of Orthopedics, UHealth Sports Medicine Institute, University of Miami, Miller School of Medicine, Miami, FL, United States; ^2^Diabetes Research Institute & Cell Transplantation Center, University of Miami, Miller School of Medicine, Miami, FL, United States

**Keywords:** MSC functionalization, MSC spheroids, MSC therapeutic properties, MSC anti-inflammatory properties, mesenchymal stem cell manufacturing

## Abstract

Mesenchymal stem/stromal cell (MSC) exist within their *in vivo* niches as part of heterogeneous cell populations, exhibiting variable stemness potential and supportive functionalities. Conventional extensive 2D *in vitro* MSC expansion, aimed at obtaining clinically relevant therapeutic cell numbers, results in detrimental effects on both cellular characteristics (e.g., phenotypic changes and senescence) and functions (e.g., differentiation capacity and immunomodulatory effects). These deleterious effects, added to the inherent inter-donor variability, negatively affect the standardization and reproducibility of MSC therapeutic potential. The resulting manufacturing challenges that drive the qualitative variability of MSC-based products is evident in various clinical trials where MSC therapeutic efficacy is moderate or, in some cases, totally insufficient. To circumvent these limitations, various *in vitro/ex vivo* techniques have been applied to manufacturing protocols to induce specific features, attributes, and functions in expanding cells. Exposure to inflammatory cues (cell priming) is one of them, however, with untoward effects such as transient expression of HLA-DR preventing allogeneic therapeutic schemes. MSC functionalization can be also achieved by *in vitro* 3D culturing techniques, in an effort to more closely recapitulate the *in vivo* MSC niche. The resulting spheroid structures provide spatial cell organization with increased cell–cell interactions, stable, or even enhanced phenotypic profiles, and increased trophic and immunomodulatory functionalities. In that context, MSC 3D spheroids have shown enhanced “medicinal signaling” activities and increased homing and survival capacities upon transplantation *in vivo*. Importantly, MSC spheroids have been applied in various preclinical animal models including wound healing, bone and osteochondral defects, and cardiovascular diseases showing safety and efficacy *in vivo*. Therefore, the incorporation of 3D MSC culturing approach into cell-based therapy would significantly impact the field, as more reproducible clinical outcomes may be achieved without requiring *ex vivo* stimulatory regimes. In the present review, we discuss the MSC functionalization in 3D settings and how this strategy can contribute to an improved MSC-based product for safer and more effective therapeutic applications.

## MSC Therapeutic Properties in Conventional 2D Cultures

Mesenchymal stem/stromal cells (MSC) are non-hematopoietic cells first isolated from the bone marrow tissue by Friedenstein et al. (1974), and thereafter from various other connective tissues and biological fluids including fat pad (Dragoo et al., 2003), adipose (Zuk et al., 2002), synovium (De Bari et al., 2001), synovial fluid (Jones et al., 2008), and umbilical cord (Weiss and Troyer, 2006). The perceived advantage of MSC as cell therapy is associated with their ease of isolation and high proliferative capacity while retaining their stemness *in vitro*, but most importantly their paracrine immunomodulatory and trophic (i.e., angiogenic, anti-fibrotic, anti-apoptotic, and mitogenic) actions *in vivo*. On this basis, MSC “medicinal signaling” activities (Caplan, 2017) exploit their environmental sensory capacity and by secretion of modulatory mediators induce the restoration of the distorted local homeostasis of the target tissue. The immunomodulatory effects of MSC are mediated by secreted bioactive molecules (i.e., IDO, PGE2, TGFβ, IGF, and IL-10), and by cell–cell contact affecting both innate and adaptive immunity (Waterman et al., 2010; Caplan and Correa, 2011; Singer and Caplan, 2011; Bernardo and Fibbe, 2013; Krampera et al., 2013; Uccelli and Rosbo, 2015; Kouroupis et al., 2017, 2018). The trophic effects are mediated by several bioactive molecules resulting in anti-apoptotic [VEGF, HGF, IGF-I, stanniocalcin-1 (STC-1), TGF-β, and GM-CSF] and mitotic (SCF, LIF, M-CSF, SDF-1, and angiopoietin-1) effects on tissue-intrinsic progenitors (da Silva Meirelles et al., 2009). Most importantly, MSC support the new vessel formation not only by functioning as pericytes and stabilizing newly formed vasculature (Sorrell et al., 2009) but also by secreting ECM molecules and angiogenic factors (VEGF, IGF-1, PIGF, MCP-1, bFGF, and IL-6) (da Silva Meirelles et al., 2009).

As reviewed in Kouroupis et al. (2017), MSC therapeutic usage *in vivo* in both autologous and allogeneic settings is safe due to their immunoevasive characteristics, and therefore, even multiple infusions of allogeneic MSC do not elicit a strong immune response that can lead to rejection progression (Koç et al., 2002; Aggarwal and Pittenger, 2005; Ringden et al., 2006; Le Blanc et al., 2008; Pittenger et al., 2019). Over the past 30 years, the safety profile of MSC has been clearly demonstrated in clinical trials to treat multiple clinical indications, with efficacy starting to produce encouraging results in some of them. To date, more than 10,000 patients have been treated as part of clinical trials, with 188 phase 1 or phase 2 trials completed and 10 trials advanced to phase 3.^1^ However, to obtain clinically relevant cell numbers, therapeutic protocols usually require MSC extensive *in vitro* 2D expansion resulting in MSC products with limited stem cell potency and, as a result in some cases, only moderate or inconsistent effectiveness to treat various clinical indications. Also, according to previous studies, MSC isolated from different tissue sources demonstrate similar, but not identical, functional capacity (Guilak et al., 2010; Moretti et al., 2010; Hass et al., 2011). Efficacy and reproducibility of MSC therapies are not only affected by the composition of the cell preparation but also by the functionality of the infused MSC to consistently home and engraft within dysregulated tissues, and subsequently to predictably exert their therapeutic effects by inducing and/or modifying specific host responses. To circumvent these limitations, various *in vitro/ex vivo* techniques have been applied to manufacturing protocols to induce specific features, attributes, and functions in expanding cells. On this basis, MSC functionalization can be achieved by *in vitro* 3D culturing techniques, in an effort to more closely recapitulate the *in vivo* 3D MSC niche and therefore preserve or enhance cellular phenotypes that result in improved *in vivo* therapeutics.

## MSC Spheroid Formation and Structure

Adult MSC possesses a remarkable ability to coalesce and assemble in tri-dimensional (3D) structures, reminiscent of their innate aggregation as limb cell precursors in the mesenchymal condensation during early skeletogenesis. In that context, 3D organoid formation *in vitro* closely recapitulates the *in vivo* MSC niche by providing spatial cell organization with increased cell–cell interactions.

**FIGURE 1:**
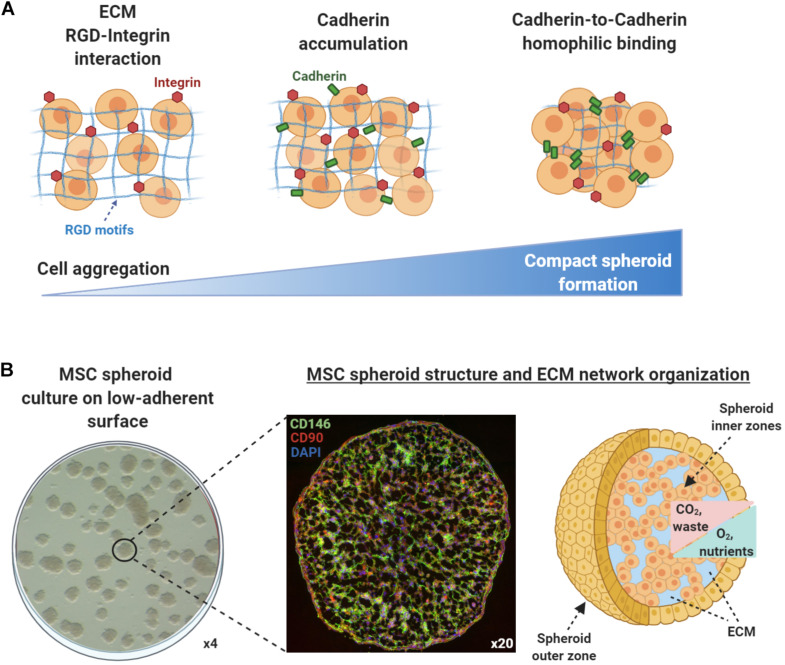
Mesenchymal stem/stromal cell (MSC) spheroids formation process and structure. **(A)** Cell aggregation and spheroid formation involving three phases. Initially, cells form loose aggregates via the tight binding of extracellular matrix arginine–glycine–aspartate (RGD) motifs with membrane-bound integrin. Due to increased cell–cell interactions, *cadherin* gene expression levels are upregulated and cadherin protein is accumulated on the cell membrane. In the later phase, homophilic cadherin-to-cadherin binding induce the formation of compact cell spheroids from cell aggregates. **(B)** Methylcellulose-based technique can be used to generate viable MSC spheroids on low-attachment gas-permeable plates (left panel). Generated MSC spheroids show stable immunophenotypic profile by expressing high levels of the pericytic marker CD146 (green) and MSC-related marker CD90 (red) (middle panel) (unpublished data). Structurally, based on their size and abundance of nutrients and oxygen *in vitro*, MSC spheroids can be divided into zones (outer and inner). The nutrients, oxygen, and waste concentration gradients within the spheroids should be always taken into consideration when selecting the optimal technique to generate spheroids *in vitro* in order to achieve increased spheroid functionality in *in vivo* settings (right panel).

According to the differential adhesion hypothesis that was first introduced in the 1960s, the cell movement and cell aggregation phenomena present in self-assembly processes are driven by differential cadherin expression levels and guided by the reduction of adhesive-free energy as cells tend to maximize their mutual binding (Foty and Steinberg, 2005). In general, cell aggregation and subsequent multicellular spheroid formation processes involve three phases (Figure 1A). Initially, cells form loose aggregates via the tight binding of extracellular matrix arginine–glycine–aspartate (RGD) motifs with membrane-bound integrin. As a result of the increased cell–cell interactions, *cadherin* gene expression levels are upregulated, whereas cadherin protein is accumulated on the cell membrane. During the later phase, homophilic cadherin-to-cadherin binding induce the formation of compact cell spheroids from cell aggregates. The extracellular matrix proteins and cadherin type and concentration are variable between different cell types, whereas other intercellular proteins such as connexin, pannexins, and actin cytoskeleton filaments play crucial roles in cell–cell interactions and subsequent multicellular cell spheroid formation (reviewed in Cui et al., 2017). Structurally, based on their size and abundance of nutrients and oxygen *in vitro*, most multicellular spheroids can be divided into three zones (Mueller-Klieser, 1984; Alvarez-Pérez et al., 2005; Curcio et al., 2007; Figure 1B). The outer asynchronously proliferative zone contains cells with intact nuclei that are proliferative with active metabolism. The intermediate zone contains cells with shrunk nuclei that are in quiescent state possessing minimum metabolic activities. Usually depending on the spheroid size, the inner necrotic zone contains cells with disintegrated nuclei that are senescent/apoptotic due to limited nutrients and oxygen influx (hypoxia) in the spheroid core. The inner necrotic zone is formed as the diffusion limitation of most molecules in spheroids is 150–200 μm, and as a result, metabolic wastes are gradually accumulating within the spheroid core. Additionally, Curcio et al. (2007) indicated that aggregates of 200-μm diameter or greater show severe oxygen limitation in the most part of their dimensions, and Alvarez-Pérez et al. (2005) related drastic intra-spheroidal pH alterations to spheroid size, with spheroids of 600-μm diameter or greater showing acidic necrotic core. Based on these findings, a three-part spheroid zonation is highly dependent on cell aggregation size and microenvironment conditions, whereas a 200-μm diameter can be putatively considered a reliable size threshold for limited/diminished inner necrotic core zone formation. Therefore, the nutrients, oxygen, and waste concentration gradients within the spheroids should be always taken into consideration when selecting the optimal technique to generate spheroids *in vitro* in order to achieve increased spheroid functionality in *in vivo* settings.

The organization of MSC in 3D spheroids result in altered cell morphology, cytoskeleton rearrangement, and polarization due to the cell–cell and cell–extracellular matrix interactions within the spheroid structure. Additionally, 3D cultures account for the established reduction in size of individual MSC (about 0.25–0.5 the volume of an average 2D cultured cell) (Bartosh et al., 2010). Specifically, studies showed that individual MSC strain is increased within the spheroid structure and equally dispersed in all cell dimensions (a Young’s Modulus of approximately 60 Pascal), whereas overall MSC tension is greater in the outer zone compared with the inner zone of spheroids. These tension differences affect MSC morphology and polarization resulting in a more flattened morphology and high integrin expression for outer zone MSC and a more irregular morphology with high cadherin expression for the inner zone MSC (Baraniak et al., 2012; Sart et al., 2014). On this basis, Lee et al. (2012) indicated E-cadherin as the main calcium-dependent adhesion molecule that plays a crucial role in MSC spheroid formation *in vitro*. During spheroid formation, E-cadherin activation and cell–cell interactions regulate the proliferative and paracrine activity of MSC via the ERK/AKT signaling pathway (Lee et al., 2012). Importantly, studies showed that cadherins, and especially N-cadherin and OB-cadherin, are both affecting the proliferation, migration, and differentiation potential of 2D MSC cultures (Theisen et al., 2007; Xu et al., 2013). Of note, cadherin levels may be important in mediating MSC anti-inflammatory actions as reports indicated that they are crucial in the response of synovial fibroblasts to inflammation (Agarwal and Brenner, 2006; Chang et al., 2011). To this end, engineered cadherin surfaces and engineered surface microtopology have been generated to control differentiation, and cell-to-cell adhesion and signaling of 2D cultured MSC *in vitro* (reviewed in Alimperti and Andreadis, 2015). However, the inherent increased cadherin levels upon MSC spheroid formation can be directly related to increased MSC spheroid functionality *in vitro* and *in vivo*, offering an advantage over 2D MSC cultures.

Interestingly, studies showed that mild hypoxia present within the inner zones of MSC spheroids positively affect MSC survival and secretory capacity. Moreover, spheroid hypoxic microenvironment upregulate the expression of hypoxia-adaptive molecules (such as *CXCL12* and *HIF-1*α), inhibit MSC apoptosis, and increase the secretion of angiogenic and anti-apoptotic molecules including HGF, VEGF, and FGF-2 compared to 2D MSC cultures (Bhang et al., 2011). Specifically, studies showed that MSC spheroids embedded in fibrin gel secrete up to 100-fold more VEGF compared with dissociated MSC in fibrin gel (Murphy et al., 2014). Except these molecules, the angiogenic trophic enhancement is produced via the upregulation of other key angiogenic factors such as angiogenin (ANG) and angiopoietin 2 (ANGPT-2; Potapova et al., 2007; Potapova et al., 2008; Yeh et al., 2014). However, Murphy et al. (2017) reported that even though cellular metabolism decreased significantly with higher cell numbers and resultant spheroid sizes, oxygen tension show a gradient that vary less than 10% from the outer zone to the inner core even for spheroids with diameters up to 353 ± 18 μm. This indicates that increased MSC functionality within the spheroid is not oxygen gradient driven but due to increased ECM production and autocrine signaling. Overall, the advantages and disadvantages of MSC functionalization in 3D spheroids are described in Table 1.

**TABLE 1:** Advantages and disadvantages of MSC functionalization in 3D spheroids.

Advantages	Disadvantages
• Increased stability of MSC immunophenotypic and molecular profiles • Enhanced stemness features and differentiation potential • Upon infusion, enhanced survival and homing *in vivo* • Enhanced secretory profile exerting mitogenic, anti-apoptotic, angiogenic, anti-fibrotic and anti-inflammatory properties	• Size variability depending on the technique used to generate spheroids •Depending on spheroid size, nutrients, oxygen, and waste concentration gradients within the spheroids •Depending on spheroid size, necrotic spheroid core formation •Depending on the clinical needs, development of reproducible, simple and cost-effective techniques are needed for large-scale production of MSC spheroids

## Methods and Biomaterials Used to Generate MSC Spheroids *ex vivo*

Lately, standardization of MSC manufacturing has been extensively evaluated in order to translate *in vitro* and *in vivo* preclinical research into safe and effective therapeutic products. Toward this goal, the large-scale clinical-grade generation of MSC spheroids possessing enhanced functionality *in vivo* is an imminent need for various therapeutic applications. To date, various methods have been used to generate MSC spheroids including the “classic” hanging drop technique and other improved methods such as the application of low-adhesive substrates, the membrane-based aggregation, and the forced aggregation techniques (reviewed in Petrenko et al., 2017).

## Scaffold-Free Mesenchymal Stem/Stromal Cell Spheroid Culture Platforms

**FIGURE 2:**
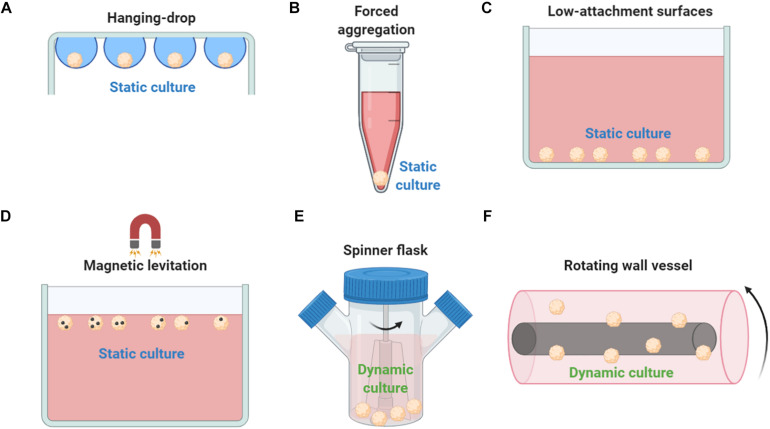
Methods used to generate MSC spheroids *ex vivo*. **(A)** In hanging drop technique, MSCs are aggregated by gravitational force. **(B)** Forced aggregation technique (or pellet culture) is used to generate MSC aggregates by gravitational force that are further induced toward 3D differentiation protocols. **(C)** Low-attachment surfaces allow the self-organization of cells into suspended spheroids. **(D)** Magnetic levitation diminish gravitational force, promotes cell–cell contact and induces cell aggregation *in vitro*. **(E)** In a spinner flask bioreactor system, cells are continuously mixed by stirring. **(F)** Rotating wall vessel technique simulates microgravity by constant circular rotation and, therefore, cells are continuously in suspension. Both dynamic culturing techniques (spinner flask and rotating wall vessel) form viable compact MSC spheroids but with altered cell size, altered phenotypic and molecular profiles, and enhanced differentiation potential compared to 2D conventional MSC cultures.

Mesenchymal stem/stromal cell spheroid culture platforms are usually trivial, rapid, and low-cost methods to generate spheroids in a non- or low-adherent environment that allows the self-organization of cells into suspended spheroids (Figures 2A–D). In the hanging drop technique, MSCs are aggregated by gravitational force but due to the absence of direct contact with solid surfaces, the composition of ECM proteins is the main factor for the regulation of spheroid microenvironment (Foty, 2011). Therefore, the hanging drop technique can generate MSC spheroids of controlled size and number; however, its main limitation is the laborious preparation of the 3D cultures that significantly limits the large-scale production of spheroids for *in vivo* applications. Using the hanging drop technique, Bartosh et al. indicated a 100-fold upregulation of anti-inflammatory (*TSG-6*) and anti-tumorigenic (*IL-24* and *TRAIL*) genes compared to 2D MSC cultures (Bartosh et al., 2010). In addition, the hanging drop technique results in higher expression of stemness markers *Oct4*, *Sox2*, and *Nanog* in MSC spheroids compared to 2D MSC cultures (Lou et al., 2016). Forced aggregation technique (or pellet culture) is also used to generate scaffold-free MSC aggregates by gravitational force that are further induced toward 3D differentiation protocols such as high-density MSC chondrogenic pellet culture (Mackay et al., 1998). A less laborious and more standardized technique is the use of low-attachment surfaces. Similar to the hanging drop technique, spontaneously secreted ECM proteins are regulating the spheroid microenvironment, however, generated spheroids show increased variability in size and morphology (Redondo-Castro et al., 2018b). Interestingly, studies showed that MSC spheroids generated on low-attachment surfaces secreted more hypoxia-induced angiogenic cytokines including VEGF, SDF, and HGF, whereas phosphorylation of Akt cell survival signaling was higher and the expression of pro-apoptotic molecules lower in MSC spheroids compared with 2D MSC cultures (Lee et al., 2016). Magnetic levitation can be used to generate MSC spheroids as by diminishing gravitational force, it promotes cell–cell contact and induces cell aggregation *in vitro*. In detail, cells are mixed with magnetic particles in culture, and cells incorporated with them can levitate due to exogenously applied magnetic field. Although preliminary studies show spheroid formation reproducibility and stable MSC spheroid phenotype, others have reported that abnormal gravity induces classic apoptotic alterations such as cell size reduction and cell membrane blebbing, reduced cell viability, nuclear chromatin condensation and margination, and increased caspase-3/7 activity (Meng et al., 2011).

Except the static techniques, various dynamic approaches have been investigated to generate MSC spheroids including spinner flask culture and rotating wall vessel techniques (Figures 2E,F). Spinner culture technique is based on a spinner flask bioreactor system where cells are continuously mixed by stirring, whereas rotating wall vessel technique simulates microgravity by constant circular rotation where cells are continuously in suspension. In a comparative study between dynamic and 2D MSC cultures, Frith et al. indicated that both spinner and rotating wall vessel dynamic cultures can form viable compact MSC spheroids showing altered cell size, altered phenotypic and molecular profiles, and enhanced osteogenic and adipogenic differentiation potential (Frith et al., 2010). Further studies showed that rotating wall vessel microgravity dramatically affect the molecular profile of MSC spheroids by upregulating genes related to adipogenic and downregulating genes related to osteogenic and chondrogenic differentiation potentials (Sheyn et al., 2010). MSC spheroid culturing in microgravity conditions results in reduced osteogenic differentiation due to decreased Collagen I gene expression and subsequent Collagen I/integrin signaling pathway activation (Meyers et al., 2004). Also, microgravity disrupts F-actin stress fibers, increase intracellular lipid accumulation, and significantly reduces RhoA activity (Meyers et al., 2005). Interestingly, others indicated that microgravity has a synergistic effect with chemical induction in stimulation of chondrogenesis mediated by p38 MAPK activation (Yu et al., 2011).

The abovementioned advantages of MSC spheroids over 2D MSC cultures make them a great candidate as building blocks for 3D bioprinting. For the large-scale manufacturing of spheroid-based tissue complexes *in vitro*, various 3D bioprinting techniques have been reported including extrusion-based bioprinting (Jakab et al., 2008; Mironov et al., 2009; Bulanova et al., 2017; Mekhileri et al., 2018), droplet-based bioprinting (Gutzweiler et al., 2017), Kenzan (Moldovan et al., 2017), and biogripper (Blakely et al., 2015; Ip et al., 2016) approaches. Studies showed that homogeneous MSC-derived cartilage spheroids with a mean diameter of 116 ± 2.8 μm can be assembled using extrusion-based bioprinting into viable cartilage constructs with stable phenotype (De Moor et al., 2020). Also, MSC-derived adipose spheroids bioprinted into a microtissue showed multilocular microvacuoles and successful differentiation toward mature adipocytes (Colle et al., 2020). However, existing 3D bioprinting techniques involve several limitations related to substantial damage to biological, structural, and mechanical spheroid properties. Recently, Ayan et al. (2020) proposed aspiration-assisted bioprinting as a novel approach for MSC spheroid assembly that causes minimal cellular damage and precisely bioprint a wide range of spheroid dimensions (ranging from 80 to 800 μm). On this basis, authors demonstrated the patterning of angiogenic sprouting spheroids and self-assembly of osteogenic spheroids. Further advancements into bioprinting field would benefit the generation of various types of MSC spheroid-derived microtissues *in vitro*.

## Scaffold-Based Mesenchymal Stem/Stromal Cell Spheroid Culture Platforms

In addition to the scaffold-free culture platforms, various scaffold-based MSC spheroid generation approaches have been proposed using both natural and synthetic biomaterials. As mentioned before, MSC spheroids can benefit the *in vivo* microenvironment primarily by their immunomodulatory and trophic actions, and secondarily (if any) by their direct differentiation toward specialized cells. The latter supports the notion that MSC spheroids should maintain their integrity in order to achieve effective cell replacement *in vivo* as biodegradation is a key factor in tissue engineering. Therefore, depending on the therapeutic application mode, biomaterial selection except from biological factors (cell adhesion, biocompatibility, etc.) should take into consideration physic-chemical (porosity to support nutrients/oxygen influx, biodegradation, etc.) parameters (Nikolova and Chavali, 2019). On this basis, even though scaffold’s topography allows seeded MSC to form a microstructured matrix within the 3D spheroid microenvironment, depending on the treated tissue’s nature, scaffold biodegradation rate should be controlled accordingly by the incorporation of chemical components that trigger gradual hydroytic degradation. However, to date, no specific studies have been performed to define if long-term maintenance of MSC spheroid structure is crucial for its therapeutic use.

Scaffold-based culture platforms using natural polymers such as agar/agarose, chitosan, and collagen can promote spheroid formation. Agar/agarose non-adherent surfaces have been used to promote MSC aggregation and spheroid formation *in vitro* (Vorwald et al., 2018). Specifically, chitosan-based substrates result in a more complex spheroid microenvironment compared to scaffold-free methods as the carbohydrate structure of chitosan is similar to the glycosaminoglycans in the ECM (Cui et al., 2017). Chitosan is a polycationic natural biocompatible polysaccharide, whereas the degree of its deacetylation can modulate the cell adhesion and spheroid formation capacity *in vitro*. On this basis, highly deacetylated chitosan substrate supports strongly the attachment and proliferation of fibroblasts (Seda Tığlı et al., 2007). Interestingly, Yeh et al. showed that MSC spheroid culturing on chitosan membranes results in increased intracellular calcium levels, whereas the calcium binding capacity of chitosan affect the cell–substrate and cell–cell interactions within the MSC spheroid. As a result, the chitosan-cultured MSC spheroids show significantly upregulated expression of calcium-, cell adhesion/migration-, and anti-inflammatory-associated genes compared to 2D MSC on tissue culture polystyrene plates (Yeh et al., 2012, 2014). Hsu and Huang showed that Wnt signaling is not only distinct in MSC spheroids compared to 2D MSC cultures but also substrate dependent. MSC spheroids derived on chitosan-activated Wnt3α-mediated canonical Wnt signaling is prone to osteogenesis, whereas MSC spheroids derived on hyaluronan-grafted chitosan activated Wnt5α-mediated non-canonical Wnt signaling that is prone to chondrogenesis (Hsu and Huang, 2013). On this basis, Huang et al. (2011) showed that MSC spheroids generated on chitosan and chitosan–hyaluronan substrates preserve the expression of stemness markers *Oct4*, *Sox2*, and *Nanog*, and increase their chondrogenic differentiation capacity. As autophagy is an important mechanism promoting cell survival, a study showed that MSC spheroids derived on chitosan respond to environmental stress (H_2_O_2_ treatment) by upregulating autophagy-related markers in a calcium-dependent manner (Yang et al., 2015). This effect is important as it may increase the MSC spheroid survival and therapeutic efficacy in *in vivo* settings. Interestingly, nanomagnetically levitated MSCs cultured as spheroids within type I collagen gels preserve their quiescent phenotype indicated by the expression of *STRO-1* and *Nestin*, whereas in response to co-culture wounding, they are capable of migrating to the wound site and differentiate accordingly (Lewis et al., 2016).

Polymers and chemically modified polymers have been extensively investigated for the development of novel biomaterials with good physic-chemical properties and biocompatibility. On this basis, MSC spheroid generation has been performed on various synthesized polymer substrates such as polycaprolactone, micropatterned poly(ethylene glycol), poly(L-glutamic acid)/chitosan, and methylcellulose. In one study, Messina et al. (2017) showed that fibroblast, myoblast, and neural cell spheroids on polymeric membranes possess high biological activity in terms of oxygen uptake, whereas they undergo faster fusion and maturation on polycaprolactone than on agarose substrates. Also, Wang W. et al. (2009) showed improved adipogenic and osteogenic differentiation capacity of MSC spheroids generated on micropatterned poly(ethylene glycol) substrates. Microarray analysis indicated not only the upregulation of genes related to adipogenesis and osteogenesis but also the downregulation of genes related to MSC stemness such as the mesoderm–specific transcript (*MEST*) and the mesenchymal stem cell specific marker (*THY1*) (Wang W. et al., 2009). Similarly, Zhang et al. (2015) indicated that MSC spheroids generated on poly(L-glutamic acid)/chitosan substrate show increased chondrogenic differentiation capacity by increased GAGs and COLII, and decreased COLI deposition during *in vitro* chondrogenic induction.

Methylcellulose, an ether derivative of cellulose, which is synthesized by the replacement of hydrogen atoms from hydroxy groups with methyl groups, has been recently used to generate successfully MSC spheroids *in vitro*. Deynoux et al. (2020) showed that methylcellulose allows MSC spheroid formation within 24 h, which tends to shrink in size partially due to the balance between proliferation and cell death triggered by hypoxia and oxidative stress up to 3 weeks *in vitro*. Similar to methylcellulose-based technique published by Markou et al. (2020), we have generated successfully viable MSC spheroids in a gas-permeable plate system that possess stable phenotypic and molecular profiles, and increased functionality both *in vitro* and *in vivo* (Kouroupis et al., 2021). The usage of this system is aimed to ensure uniform oxygenation throughout the MSC spheroid culture, as it is based on previous reports demonstrating that in gas-permeable plates 3D cell structures efficiently receive air from both the top (after diffusion through the medium) and the bottom (after diffusion across permeable membrane) of the culture (Fraker et al., 2007, 2013; Cechin et al., 2014). These reports show that MSC spheroid generation on synthesized substrates can dramatically affect their stemness and multipotential differentiation capacities *in vitro*.

## Culture Medium Effects on Mesenchymal Stem/Stromal Cell Spheroids

With the exception of the scaffold-free or scaffold-based culture platforms, reports showed that culture medium composition strongly affect the spheroid formation progression and MSC spheroid functionality *in vitro*. To date, most studies use fetal bovine serum (FBS)-based media to generate spheroids *in vitro*. However, safety concerns have been raised regarding FBS usage for the manufacturing of MSC products for clinical applications, most of them related to prion exposure risk, toxicological risk, and immunological risk (Mendicino et al., 2014; Karnieli et al., 2017). Regulatory-complaint xeno-free media such as chemically defined formulations and human platelet lysate (hPL) are promising alternatives to generate clinically relevant cell numbers and to preserve or even enhance the MSC functionality *in vitro* prior to their *in vivo* application (Doucet et al., 2005; Centeno et al., 2008; Jung et al., 2010; Kouroupis et al.,2020a,b). On this basis, Ylostalo et al. (2014) showed that MSCs cannot condense into tight spheroids when cultured in several commercial stem cell media and only chemically defined formulation supplemented with human serum albumin (HSA) can result in compact MSC spheroids with high viability and enhanced anti-inflammatory secretory profile. Importantly, MSC spheroids generated with HAS supplementation show increased anti-inflammatory capacity when co-cultured with lipopolysaccharide-stimulated macrophages *in vitro* (Ylostalo et al., 2014). In contrast, another study indicated that MSC spheroids generated in FBS-based medium show low or no proliferation but increased paracrine secretory profile (PGE2 and IDO), whereas MSC spheroids generated in xeno-free medium show significant proliferative capacity but low paracrine secretory profile (Zimmermann and McDevitt, 2014).

Overall, further investigations have to be performed in order to optimize the *in vitro* culturing conditions for the standardization and reproducibility of MSC spheroid therapeutic potential. Most importantly, challenges still exist related to the generation of clinically relevant cell numbers in 3D cultures and the qualitative assessment of the generated MSC spheroids using conventional methods. Specifically, the less laborious dynamic approaches, such as the spinner flask culture and the rotating wall vessel techniques, offer a viable solution to generate large MSC spheroid numbers; however, novel bioreactor systems are needed to additionally monitor and control all culture environmental variables (temperature, gas exchange, pH, and metabolite levels) (de Bournonville et al., 2019). Similar to 2D MSC cultures, qualitative evaluation of MSC spheroids requires their phenotypic protein profiling using fluorescent microscopy and flow cytometry methods. Fluorescent imaging is often laborious for xyz images and represent only a fraction of MSC spheroid cultures, whereas flow cytometry requires the enzymatic/mechanical dissociation of the spheroids to a single cell, usually disrupting important sensitive phenotypic attributes (CD146 immunomodulation-related marker). Furthermore, comparative preclinical studies are needed to evaluate how different MSC spheroid generation platforms *in vitro* are affecting the therapeutic outcomes upon their implantation or infusion *in vivo*.

## Anti-Inflammatory Properties of Mesenchymal Stem/Stromal Cell Spheroids

**FIGURE 3:**
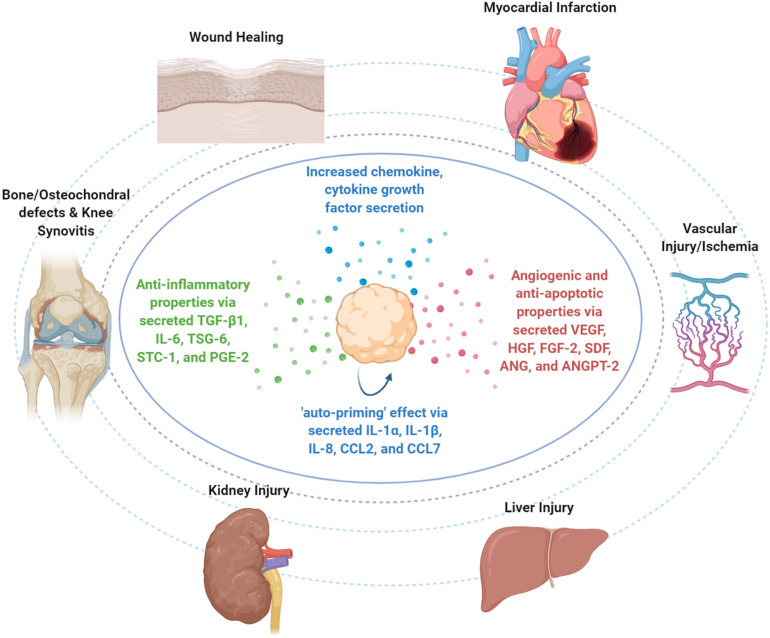
Therapeutic properties of MSC spheroids *in vivo*. Upon infusion *in vivo*, MSC spheroid “medicinal signaling” activities are exerted by the paracrine secretion of modulatory mediators that possess immunomodulatory and trophic (i.e., angiogenic, anti-fibrotic, anti-apoptotic, and mitogenic) actions. MSC spheroids have been safely and effectively applied in various preclinical animal models for the treatment of skin wounds, myocardial infarction, vascular injury/ischemia, liver injury, kidney injury, bone and osteochondral defects, and knee synovitis.

In MSC spheroid settings, their enhanced anti-inflammatory effects have been mainly attributed to high expression of TGF-β1, IL-6, TSG-6, stanniocalcin (STC-1), and PGE-2 anti-inflammatory molecules (Bartosh et al., 2010; Ylöstalo et al., 2012; Zimmermann and McDevitt, 2014; Figure 3). Specifically, Bartosh et al. showed that BM-derived MSC spheroid increased secretion of anti-inflammatory TSG-6 and STC-1 results in reduced TNFα expression and secretion by LPS-stimulated macrophages in MSC spheroid/macrophages co-cultures *in vitro*. In a mouse zymosan-induced peritonitis model, intraperitoneal injection of 1.5 × 10^6^ BM-derived MSC spheroids for a 6-h time-frame resulted in decreased protein content and volume of the lavage fluid, neutrophil activity, and decreased levels of TNFα, IL-1β, CXCL2/MIP-2, and PGE2. Also, MSC spheroid injection significantly decreased the serum levels of plasmin activity, an inflammation-related protease that is inhibited by secreted TSG-6 (Bartosh et al., 2010). Importantly, *in vitro* studies showed that BM-derived MSC spheroid conditioned medium affect LPS-stimulated macrophages not only by inhibiting the secretion of pro-inflammatory cytokines TNFα, CXCL2, IL-6, IL12-p40, and IL-23 but also by increasing the secretion of anti-inflammatory cytokines IL-10 and IL1-Ra and the expression of M2-polarization CD206 marker. The main anti-inflammatory molecule secreted in the conditioned medium was PGE2, whereas its production is dependent on caspase activity and NFkB activation in MSC spheroids (Ylöstalo et al., 2012).

Upon MSC homing to the target site and depending on the molecular composition of the local microenvironment, they exhibit a therapeutic responsive polarization into either anti-inflammatory (MSC-2) or pro-inflammatory (MSC-1) phenotypes. Interestingly, studies showed that except for the abovementioned secreted molecules with anti-inflammatory effects, MSC spheroids increase the secretion of pro-inflammatory cytokines (including IL-1α, IL-1β, and IL-8) and chemokines (including CCL2 and CCL7) (Potapova et al., 2007; Bartosh et al., 2010, 2013; Yeh et al., 2014) that contribute in the inflammatory cell recruitment locally and putatively in the overall inflammatory response of the host. However, Bartosh et al. showed that BM-derived MSC assembly into MSC spheroids triggers the caspase-dependent IL-1 signaling and activates the expression of IL-1 in an autocrine secretion manner, resulting in an “auto-priming” effect (Figure 3). In MSC spheroids, the increased PGE2 secretion was related to activation of both caspase-dependent IL-1 and Notch signaling pathways, whereas TSG-6 and STC-1 secretion was related only to caspase-dependent IL-1 signaling activation (Bartosh et al., 2010). Collectively, MSC priming by paracrine and/or autocrine pro-inflammatory modes is a prerequisite in order to acquire their anti-inflammatory MSC2 phenotype and exert strong anti-inflammatory effects *in vivo*.

As reviewed in Kouroupis et al. (2018), several studies indicate that activation of specific Toll-like receptors (TLRs) in MSC *in vitro* prior to infusion *in vivo* has a profound effect on MSC functionalization toward immunomodulatory phenotype. However, Redondo-Castro et al. (2018a) reported that IL-1 stimulation of BM-derived MSC spheroids resulted in significantly increased expression of IL1-Ra, VEGF, and G-CSF molecules without anti-inflammatory effects on LPS-treated microglial cells in co-cultures. These discrepancies of the data underline the necessity for optimization of the priming methods and culture conditions. Previous studies showed that MSC immunomodulatory factor secretion is strongly affected by the composition of the culture medium (Zimmermann and McDevitt, 2014). In 2D culture settings, BM-derived and adipose-derived MSC cultured with FBS or hPL showed differences in expression of immunomodulatory and adhesion molecules, with adipose-derived MSC being more potent functionally in inhibiting T-cell proliferation (Menard et al., 2013). Similarly, in two studies, Kouroupis et al. (2020a; 2020b) indicated that fat pad-derived (IFP) MSCs when cultured in regulatory-compliant conditions *in vitro* are superior functionally in Substance P degradation and T-cell proliferation inhibition compared to FBS-grown MSC. In an acute synovitis rat model, IFP-MSC intra-articular injection *in vivo* reversed more effectively signs of synovitis and IFP fibrosis when they were cultured under regulatory-compliant conditions (Kouroupis et al.,2020a,b). In 3-D settings, MSC spheroids cultured in serum and animal component-free chemically defined medium had less secretion of IDO, PGE2, TGF-β1, and IL-6 immunomodulatory factors compared to the typical MSC cultures supplemented with FBS (Zimmermann and McDevitt, 2014). In order to overcome these hurdles, Ylostalo et al. proposed specific protocols to efficiently prime MSCs in 3-D settings and preserve their robust anti-inflammatory properties under chemically defined xeno-free conditions (Ylostalo et al., 2017).

Overall, further studies are required to address the effects of pro-inflammatory cytokines and culturing conditions on anti-inflammatory properties of MSC spheroids *in vitro* and *in vivo*.

## Therapeutic Properties of Mesenchymal Stem/Stromal Cell Spheroids in Preclinical Animal Models

With exception to the therapeutic safety that most MSC clinical trials are investigating for various clinical disorders,^2^ crucial factors that affect the therapeutic efficiency are MSC homing to target tissues and subsequent MSC survival *in vivo*. It cannot be overlooked that initial outcomes from many of such studies revealed that MSC therapies show a significant degree of variability with cases of non-reproducible clinical data. The inconsistent evidence potentially relates not only to intrinsic differences in the cell-based products used but importantly related with their *in vivo* fate upon implantation or infusion [parameters affecting MSC functionalization *in vitro* and *in vivo* are reviewed in Kouroupis et al. (2018)]. On this basis, a pioneering study showed that 5.0 × 10^5^ BM-MSC injected into the left ventricle of uninjured mouse heart can effectively engraft the myocardium; however, only 0.44% of the MSCs could be identified after 4 days of injection (Toma et al., 2002). In addition, Toma et al. showed that 92 ± 7% of intraarterially injected MSC in rats are entrapped in the microvasculature (Toma et al., 2009). Collectively, even though long-term engraftment seems not to be a prerequisite for MSC reparative effects *in vivo*, their initial homing and survival is a crucial factor affecting the therapeutic outcomes. In that context, 3D spheroid formation *in vitro* closely recapitulates the *in vivo* MSC niche by providing spatial cell organization with increased cell–cell interactions that protect MSC viability and intrinsic properties. For example, in a mouse model of hind limb ischemia, MSC spheroid transplantation improved its survival compared to MSC suspension, by suppressing a key apoptotic signaling molecule (Bax), while activating anti-apoptotic signaling (BCL-2; Bhang et al., 2012). These positive effects can also be attributed to improved resistance to oxidative stress-induced apoptosis exerted by hypoxia-induced genes (e.g., VEGF-A, HIF-1^α^, and MnSOD), elevated by the hypoxic conditions at the spheroid core (Potapova et al., 2007; Zhang et al., 2012).

Mesenchymal stem/stromal cell-based spheroids have been applied in various preclinical models including wound healing (Amos et al., 2009; Zhang et al., 2012; Hsu and Hsieh, 2015), bone and osteochondral defects (Ma et al., 2011; Suzuki et al., 2012; Suenaga et al., 2015), knee synovitis (Kouroupis et al., 2021), and cardiovascular diseases (Wang C.-C. et al., 2009; Emmert et al., 2013a) (Figure 3).

## Wound Healing

To date, three separate studies applied MSC spheroids for wound healing in a model of diabetic healing impaired (leptin receptor-deficient) mice (Amos et al., 2009), in chemotherapy-induced oral mucositis (Zhang et al., 2012), and in a rat skin repair model (Hsu and Hsieh, 2015). In a pioneering study, Amos et al. investigated the applicability of MSC spheroids to treat chronic wounds such as diabetic ulcers, which remain a significant health burden for diabetic patients. In detail, full-thickness dermal wounds (approximately 78.5 mm^2^ area) were generated in leptin receptor-deficient mice and treated with a total of 350,000 adipose-derived MSC per wound organized in multiple separate spheroids. Interestingly, for a 12 day time-frame, MSC spheroids resulted in significantly greater rate of wound closure compared to wounds treated with MSC suspension. This outcome may be attributed to higher expression of ECM genes (*tenascin C*, *Collagen VI*α*3*, and *fibronectin*) and higher secretion of soluble factors (HGF, MMP-2, and MMP-14) in MSC spheroid compared to MSC suspension cultures *in vitro* (Amos et al., 2009). Zhang et al. (2012) intravenously infused 1 × 10^6^ gingiva-derived MSC spheroids or MSC suspension to a 5-fluorouracil-induced oral mycositis mouse model. On day 7, results indicated that MSC spheroids can reverse body weight loss and promote the regeneration of damaged epithelial lining of the mucositic mouse tongues. Interestingly, authors reported that MSC spheroids are capable of increased homing/engrafting to mucositic tongues due to their enhanced CXCR4 expression and may potentially trans-differentiate into epithelial cells via mesenchymal–epithelial transition *in vivo* (Zhang et al., 2012). These data indicate the potential use of MSC spheroids to alleviate the oral mucositis side-effect post-chemotherapy in cancer patients. In another rat skin wound healing model, 1 × 10^5^ adipose-derived MSC spheroids or MSC suspension were applied to 15 mm × 15 mm wounds and covered with hyaluronan gel/chitosan sponge to maintain a moist environment. On day 8, results showed that the MSC spheroid group showed faster wound closure and significantly higher ratio of angiogenesis compared with the MSC suspension group. *In vivo* tracking of fluorescently labeled MSCs showed close localization of MSC spheroids to microvessels, suggesting enhanced angiogenesis through paracrine effects. Moreover, MSC spheroid increased engrafting and angiogenesis effects may be attributed to the high expression of cytokine genes (*FGF-1*, *VEGF*, and *CCL2*) and migration-related genes (*CXCR4* and *MMP-1*) (Hsu and Hsieh, 2015). Collectively, in all cases, MSC spheroids provide better therapeutic efficacy compared with traditional MSC suspension in wound healing.

## Bone/Osteochondral Defects and Synovitis

Studies showed that bone/osteochondral defects and knee synovitis can be treated by MSC spheroids. In a delicate study, Sekiya’s group generated a full-thickness (5 mm × 5 mm wide, 1.5 mm deep) osteochondral defect rabbit model, and defects were treated with different doses of synovium-derived MSC spheroids (containing 2.5 × 10^5^–20 × 10^6^ MSC/defect) (Suzuki et al., 2012). Post-implantation MSC spheroids could attach to the osteochondral defects by surface tension, whereas at 12 weeks, MSC spheroids containing 2.5 × 10^6^ MSC showed the highest safranin-O-positive area ratio and resulted in regenerated cartilage with thickness similar to the neighboring healthy cartilage. Interestingly, authors reported that MSC spheroids with high cell densities result in failed defect repair and fibrous tissue formation possibly due to cell death and nutrient deprivation effects (Suzuki et al., 2012). In a calvarial bone defect (8 mm wide) rat model, Suenaga et al. treated the rat defects using three different conditions, 3.0 × 10^7^ BM-MSC spheroids, β-TCP granules, or BM-MSC spheroids coated with β-TCP granules. Eight weeks post-implantation, MSC spheroids resulted in full-thickness bone formation with evident vascularization. In contrast, the other two groups had only minimal or non-uniform bone formation at the implanted sites, indicating that β-TCP restricts the bone regenerative capacity of MSC spheroids (Suenaga et al., 2015). Recently, Yanagihara et al. (2018) treated 4 mm wide femoral bone defects in rats with 2.4 × 10^6^ Runx2-transfected MSC spheroids or Runx2-transfected MSC suspension embedded in collagen scaffolds. On day 35, MSC spheroids showed faster bone regeneration compared with MSC suspension and non-transfected MSC, whereas enhanced MSC spheroid migration to the defect sites was correlated with higher expression levels of migration-related genes *CXCR4* and *Integrin*α*2* (Yanagihara et al., 2018). Recently, in a mono-iodoacetate acute synovial/IFP inflammation rat model, Kouroupis et al. intraarticularly injected 5.0 × 10^5^ infrapatellar fat pad MSC (IFP-MSC) spheroids. Twenty-five days post-infusion, IFP-MSC spheroids effectively degraded Substance P and resolved inflammation and fibrosis of synovial membrane and fat pad tissues in the rat knee. Interestingly, IFP-MSC intraarticular injection not only results in anti-inflammatory and anti-fibrotic effects but also showed strong anabolic/cartilage protective effects. Specifically, in the IFP-MSC spheroid cohort, cartilage integrity was preserved intact up to 28 days (Kouroupis et al., 2021). To conclude, MSC spheroids exert anti-inflammatory/anti-fibrotic effects and are effective for promoting both bone and osteochondral defect regeneration.

## Myocardial Infarction

Intramyocardial transplantation of MSC spheroids in rat (Wang C.-C. et al., 2009; Lee et al., 2012; Liu et al., 2013) and porcine (Emmert et al., 2013b) myocardial infarction models resulted in greater heart function improvement compared with MSC suspensions. In an acute myocardial infarction rat model, Wang C.-C. et al. (2009) performed intramyocardial injection of 5.0 × 10^5^ BM-derived MSC spheroids or MSC suspension and evaluated the echocardiography and catheterization measurements 4, 8, and 12 weeks post-operatively. The results showed superior heart function and stimulation of significant increase in vascular density for the MSC spheroid group (Wang C.-C. et al., 2009). In a delicate study, *in vivo* tracking of Dil-labeled UC-derived MSC spheroids showed that they can be differentiated into endothelial and cardiomyocyte cells at 4 weeks post-intramyocardial injection in a rat myocardial infarction model. At 7 weeks, the therapeutic efficacy of UC-derived MSC spheroids is superior to MSC suspension in post-infarction left ventricular remodeling (Lee et al., 2012). Importantly, Liu et al. (2013) showed that adipose-derived MSC spheroids generated on chitosan membranes show a 20-fold increase in cardiac marker gene expression (*Gata4*, *Nkx2-5*, *Myh6*, and *Tnnt2*) compared with MSC suspension cultures. In a similar approach, intramyocardial injection of 1 × 10^7^ adipose-derived MSC spheroids in a rat myocardial infarction model showed better functional recovery compared with MSC suspensions after 12 weeks (Liu et al., 2013). Interestingly, a previous study indicated that intramyocardial injection of MSC spheroids consisting of adipose-derived MSC/human umbilical vein endothelial cells results in low arrhythmogenic potential but no further beneficial effects compared to the untreated group in a rat myocardial infarction model (Kolettis et al., 2018). In a larger animal model study, adipose-derived MSC were first labeled with micron-sized iron oxide particles, and then 2 × 10^7^ MSC spheroids or MSC suspension were intra-myocardial injected in the porcine-infarcted myocardium. Moreover, the MSC spheroid engrafted successfully in 88.8% of animals keeping intact their micro architecture *in vivo*, whereas no arrhythmogenic, embolic, or neurological events occurred in the treated groups for up to 5 weeks follow-up (Emmert et al., 2013b). Therefore, preclinical studies established the feasibility, safety, and beneficial effects of intra-myocardial injected MSC spheroids in infarcted myocardium.

## Neovascularization and Ischemia

In conjunction with the beneficial trophic effects of MSC spheroids toward infarcted myocardium, their applicability has been also investigated for neovascularization *in vivo*. In a mouse hind limb ischemia model, 1.0 × 10^7^ cord-blood MSC spheroid intramuscular injection significantly increased the number of microvessels and αSMA-positive vessels, resulting in decreased fibrosis in the ischemic region, and attenuated limb loss and necrosis. In comparison, the MSC spheroid group showed a limb salvage rate of 75%, whereas the MSC suspension group resulted in limb salvage rate of only 12.5% (Bhang et al., 2012). Additionally, Lee et al. (2016) showed that intramuscular injected adipose-derived MSC spheroids showed better proliferation than MSC suspension in the ischemic region, an effect that can be attributed to an increased expression of the proliferation marker PCNA. Therefore, MSC spheroids promote vascularization through secretion of angiogenic cytokines, preservation of ECM, and regulation of apoptotic signals.

## Liver and Kidney Disease

The potential of MSC spheroids has been also investigated in liver regeneration and kidney injury models. For liver regeneration, two animal models have been tested for hepatectomy and CCl4-induced acute liver failure. In a pioneering study, Liu and Chang (2006) injected intraperitoneally 3 × 10^7^ BM-MSC or hepatocytes in alginate–polylysine–alginate spheroids or suspension formats to treat 90% of hepatectomized rats. Up to day 14, in the BM-MSC spheroid, hepatocyte spheroid, and hepatocyte suspension groups, most rats survived (83–100%) and showed increased liver wet weight. Interestingly, these beneficial effects could be attributed to the increased expression in MSC spheroids of hepatocyte markers cytokeratin 8, cytokeratin 18, albumin, and α-fetoprotein (Liu and Chang, 2006). In an improved approach, 3 × 10^7^ BM-MSC spheroids or MSC suspension were intrasplenically injected to treat 90% of hepatectomized rats. On day 14, survival rate in MSC spheroid group was prolonged by almost 70% compared with the MSC suspension group via the secretion of hepatotrophic factors such as HGF and IL-6 into the liver. Of note, authors reported that implanted MSC may transdifferentiate into hepatocyte-like cells *in vivo* and therefore may render spleen as an ectopic functional liver support (Liu and Chang, 2009, 2012). This hypothesis has to be further investigated as MSC differentiation toward endodermal fate has not been widely established. In a CCl4-induced acute liver failure mouse model, 1 × 10^6^ UC-MSC spheroids or MSC suspension were infused via the tail vein and, at day 2, resulted in liver injury attenuation. Specifically, MSC spheroids could promote IL-6 and IFN-γ secretion but suppress TNF-α serum levels, and therefore significantly reduce tissue necrosis and increase liver regeneration (Li et al., 2015). In a recent study, adipose-derived MSC spheroids have been used to treat an ischemia–reperfusion (I/R)-induced acute kidney injury rat model. Moreover, 2 × 10^6^ MSC spheroids or MSC suspension were directly injected to the kidney cortex, and renal function was investigated for a 14-day follow-up. Results indicated that MSC spheroids are more beneficial to the kidney by reduction of tissue damage, increased vascularization, and amelioration of renal function compared with MSC suspensions. In detail, the MSC spheroid group showed increased levels of VEGF, HGF, and TSG-6 cytokines, and decreased levels of creatinine and blood urea nitrogen in the serum (Xu et al., 2016). Therefore, in both liver and kidney injury animal models, MSC spheroid paracrine actions result in improved therapeutic effects characterized by reduced tissue necrosis, increased tissue regeneration, and improved organ function.

## Future Clinical Perspectives

To date, only a limited number of comparative preclinical studies have been performed between MSC spheroids and MSC suspension after 2D culture, whereas no clinical trials exist to evaluate the efficacy of MSC spheroids in clinical settings. As a result, there are no specific criteria to define when MSC spheroids would be preferable over MSC suspension to treat various clinical indications. However, it has become increasingly clear that current conventional and extensive 2D MSC culturing methods, similar to the ones used in public and commercial stem cell biobanks, even though they can ensure the generation of clinically relevant cell numbers for *in vivo* applications, cannot guarantee the preservation of MSC qualitative characteristics and their related high functionality. To circumvent these limitations, the incorporation of 3D MSC culturing approach into cell-based therapy would significantly impact the field, as more reproducible clinical outcomes may be achieved without requiring extensive *ex vivo* MSC manipulation and MSC stimulatory regimes (reviewed in Kouroupis et al., 2018). Specifically, current data indicate that MSC spheroid cultures with or without the usage of biomaterials not only preserve MSC phenotypic and molecular profiles but also significantly reinforce MSC functionality related to their immunomodulatory, anti-fibrotic, angiogenic, and trophic properties. In addition, as initial MSC homing and survival are crucial factors affecting the therapeutic outcome, 3D spheroid formation closely recapitulates the *in vivo* MSC niche, protect MSC viability, and works as a “vehicle” for their effective homing to the affected tissues upon implantation *in vivo*. On this basis, the adaptation of high-throughput regulatory-compliant and reproducible methods for MSC spheroid production would allow their use in clinical settings and contribute to an improved MSC-based product for safer and more effective therapeutic applications.

## Author Contributions

Both authors have made substantial contributions to the drafting of the article or revising it critically and to the final approval of the version to be submitted.

## Conflict of Interest

The authors declare that the research was conducted in the absence of any commercial or financial relationships that could be construed as a potential conflict of interest.

## Footnotes

1 www.clinicaltrials.gov

2 http://clinicaltrials.gov

## References

[B1] Agarwal S. K. Brenner M. B. (2006). Role of adhesion molecules in synovial inflammation. *Curr. Opin. Rheumatol.* 18 268–276. 10.1097/01.bor.0000218948.42730.39 16582691

[B2] Aggarwal S. Pittenger M. F. (2005). Human mesenchymal stem cells modulate allogeneic immune cell responses. *Blood* 105 1815–1822. 15494428 10.1182/blood-2004-04-1559

[B3] Alimperti S. Andreadis S. T. (2015). CDH2 and CDH11 act as regulators of stem cell fate decisions. *Stem Cell Res.* 14 270–282. 10.1016/j.scr.2015.02.002 25771201 PMC4439315

[B4] Alvarez-Pérez J. Ballesteros P. Cerdán S. (2005). Microscopic images of intraspheroidal pH by 1H magnetic resonance chemical shift imaging of pH sensitive indicators. *Magma* 18 293–301. 10.1007/s10334-005-0013-z 16328228

[B5] Amos P. J. Kapur S. K. Stapor P. C. Shang H. Bekiranov S. Khurgel M. (2009). Human adipose-derived stromal cells accelerate diabetic wound healing: impact of cell formulation and delivery. *Tissue Eng. Part A* 16 1595–1606. 10.1089/ten.tea.2009.0616 20038211 PMC2952117

[B6] Ayan B. Heo D. N. Zhang Z. Dey M. Povilianskas A. Drapaca C. (2020). Aspiration-assisted bioprinting for precise positioning of biologics. *Sci. Adv.* 6:eaaw5111. 10.1126/sciadv.aaw5111 PMC7060055 32181332

[B7] Baraniak P. R. Cooke M. T. Saeed R. Kinney M. A. Fridley K. M. McDevitt T. C. (2012). Stiffening of human mesenchymal stem cell spheroid microenvironments induced by incorporation of gelatin microparticles. *J. Mech. Behav. Biomed. Mater.* 11 63–71. 10.1016/j.jmbbm.2012.02.018 22658155 PMC3528787

[B8] Bartosh T. J. Ylöstalo J. H. Bazhanov N. Kuhlman J. Prockop D. J. (2013). Dynamic compaction of human mesenchymal stem/precursor cells into spheres self-activates caspase-dependent IL1 signaling to enhance secretion of modulators of inflammation and immunity (PGE2, TSG6, and STC1). *Stem Cells* 31 2443–2456. 10.1002/stem.1499 23922312 PMC3834191

[B9] Bartosh T. J. Ylostalo J. H. Mohammadipoor A. Bazhanov N. Coble K. Claypool K. (2010). Aggregation of human mesenchymal stromal cells (MSCs) into 3D spheroids enhances their antiinflammatory properties. *Proc. Natl. Acad. Sci. U.S.A.* 107 13724–13729. 10.1073/pnas.1008117107 20643923 PMC2922230

[B10] Bernardo M. E. Fibbe W. E. (2013). Mesenchymal stromal cells: sensors and switchers of inflammation. *Cell Stem Cell* 13 392–402. 10.1016/j.stem.2013.09.006 24094322

[B11] Bhang S. H. Cho S.-W. La W.-G. Lee T.-J. Yang H. S. Sun A.-Y. (2011). Angiogenesis in ischemic tissue produced by spheroid grafting of human adipose-derived stromal cells. *Biomaterials* 32 2734–2747. 10.1016/j.biomaterials.2010.12.035 21262528

[B12] Bhang S. H. Lee S. Shin J.-Y. Lee T.-J. Kim B.-S. (2012). Transplantation of cord blood mesenchymal stem cells as spheroids enhances vascularization. *Tissue Eng. Part A* 18 2138–2147. 10.1089/ten.tea.2011.0640 22559333 PMC3463282

[B13] Blakely A. M. Manning K. L. Tripathi A. Morgan J. R. (2015). Bio-pick, place, and perfuse: a new instrument for three-dimensional tissue engineering. *Tissue Eng. Part C Methods* 21 737–746. 10.1089/ten.TEC.2014.0439 25530515 PMC4499775

[B14] Bulanova E. A. Koudan E. V. Degosserie J. Heymans C. Pereira F. D. Parfenov V. A. (2017). Bioprinting of a functional vascularized mouse thyroid gland construct. *Biofabrication* 9:034105. 10.1088/1758-5090/aa7fdd 28707625

[B15] Caplan A. I. (2017). mesenchymal stem cells: time to change the name! *Stem Cells Transl. Med.* 6 1445–1451. 10.1002/sctm.17-0051 28452204 PMC5689741

[B16] Caplan A. I. Correa D. (2011). The MSC: an injury drugstore. *Cell Stem Cell* 9 11–15. 21726829 10.1016/j.stem.2011.06.008 PMC3144500

[B17] Cechin S. Alvarez-Cubela S. Giraldo J. A. Molano R. D. Villate S. Ricordi C. (2014). Influence of in vitro and in vivo oxygen modulation on β cell differentiation from human embryonic stem cells. *Stem Cells Transl. Med.* 3 277–289. 10.5966/sctm.2013-0160 24375542 PMC3952934

[B18] Centeno C. J. Busse D. Kisiday J. Keohan C. Freeman M. Karli D. (2008). Regeneration of meniscus cartilage in a knee treated with percutaneously implanted autologous mesenchymal stem cells. *Med. Hypotheses* 71 900–908. 10.1016/j.mehy.2008.06.042 18786777

[B19] Chang S. K. Noss E. H. Chen M. Gu Z. Townsend K. Grenha R. (2011). Cadherin-11 regulates fibroblast inflammation. *Proc. Natl.Acad. Sci. U.S.A.* 108:8402. 10.1073/pnas.1019437108 21536877 PMC3100978

[B20] Colle J. Blondeel P. Bruyne A. Bochar S. Tytgat L. Vercruysse C. (2020). Bioprinting predifferentiated adipose-derived mesenchymal stem cell spheroids with methacrylated gelatin ink for adipose tissue engineering. *J. Mater. Sci.* 31:36. 10.1007/s10856-020-06374-w 32206922

[B21] Cui X. Hartanto Y. Zhang H. (2017). Advances in multicellular spheroids formation. *J. R. Soc. Interface* 14:20160877. 10.1098/rsif.2016.0877 28202590 PMC5332573

[B22] Curcio E. Salerno S. Barbieri G. De Bartolo L. Drioli E. Bader A. (2007). Mass transfer and metabolic reactions in hepatocyte spheroids cultured in rotating wall gas-permeable membrane system. *Biomaterials* 28 5487–5497. 10.1016/j.biomaterials.2007.08.033 17881050

[B23] da Silva Meirelles L. Fontes A. M. Covas D. T. Caplan A. I. (2009). Mechanisms involved in the therapeutic properties of mesenchymal stem cells. *Cytokine Growth Factor Rev.* 20 419–427. 10.1016/j.cytogfr.2009.10.002 19926330

[B24] De Bari C. Dell’Accio F. Tylzanowski P. Luyten F. P. (2001). Multipotent mesenchymal stem cells from adult human synovial membrane. *Arthritis Rheumatism* 44 1928–1942. 11508446 10.1002/1529-0131(200108)44:8<1928::AID-ART331>3.0.CO;2-P

[B25] de Bournonville S. Lambrechts T. Vanhulst J. Luyten F. P. Papantoniou I. Geris L. (2019). Towards self-regulated bioprocessing: a compact benchtop bioreactor system for monitored and controlled 3D cell and tissue culture. *Biotechnol. J.* 14:1800545. 10.1002/biot.201800545 30964231

[B26] De Moor L. Fernandez S. Vercruysse C. Tytgat L. Asadian M. De Geyter N. (2020). Hybrid bioprinting of chondrogenically induced human mesenchymal stem cell spheroids. *Front. Bioeng. Biotechnol.* 8:484. 10.3389/fbioe.2020.00484 32523941 PMC7261943

[B27] Deynoux M. Sunter N. Ducrocq E. Dakik H. Guibon R. Burlaud-Gaillard J. (2020). A comparative study of the capacity of mesenchymal stromal cell lines to form spheroids. *PLoS One* 15:e0225485. 10.1371/journal.pone.0225485 32484831 PMC7266346

[B28] Doucet C. Ernou I. Zhang Y. Llense J.-R. Begot L. Holy X. (2005). Platelet lysates promote mesenchymal stem cell expansion: a safety substitute for animal serum in cell-based therapy applications. *J. Cell. Physiol.* 205 228–236. 10.1002/jcp.20391 15887229

[B29] Dragoo J. L. Samimi B. Zhu M.-L. Hame S. L. Thomas B. L. Lieberman J. R. (2003). Tissue-engineered cartilage and bone using stem cells from human infrapatellar fat pads. *J. Bone Joint Surg. Br.* 85 740–747. 12892203

[B30] Emmert M. Y. Wolint P. Wickboldt N. Gemayel G. Weber B. Brokopp C. E. (2013a). Human stem cell-based three-dimensional microtissues for advanced cardiac cell therapies. *Biomaterials* 34 6339–6354. 10.1016/j.biomaterials.2013.04.034 23727259

[B31] Emmert M. Y. Wolint P. Winklhofer S. Stolzmann P. Cesarovic N. Fleischmann T. (2013b). Transcatheter based electromechanical mapping guided intramyocardial transplantation and in vivo tracking of human stem cell based three dimensional microtissues in the porcine heart. *Biomaterials* 34 2428–2441. 10.1016/j.biomaterials.2012.12.021 23332174

[B32] Foty R. A. Steinberg M. S. (2005). The differential adhesion hypothesis: a direct evaluation. *Dev. Biol.* 278 255–263. 10.1016/j.ydbio.2004.11.012 15649477

[B33] Foty R. (2011). A simple hanging drop cell culture protocol for generation of 3D spheroids. *J. Vis. Exp.* 51 2720. 10.3791/2720 21587162 PMC3197119

[B34] Fraker C. A. Alvarez S. Papadopoulos P. Giraldo J. Gu W. Ricordi C. (2007). Enhanced oxygenation promotes beta-cell differentiation in vitro. *Stem Cells* 25 3155–3164. 10.1634/stemcells.2007-0445 17761759

[B35] Fraker C. A. Cechin S. Álvarez-Cubela S. Echeverri F. Bernal A. Poo R. (2013). A physiological pattern of oxygenation using perfluorocarbon-based culture devices maximizes pancreatic islet viability and enhances β-Cell function. *Cell Transplantation* 22 1723–1733. 10.3727/096368912X657873 23068091

[B36] Friedenstein A. J. Chailakhyan R. K. Latsinik N. V. Panasyuk A. F. Keiliss-Borok I. V. (1974). Stromal cells responsible for transferring the microenvironment of the hemopoietic tissues. Cloning in vitro and retransplantation in vivo. *Transplantation* 17 331–340. 4150881 10.1097/00007890-197404000-00001

[B37] Frith J. E. Thomson B. Genever P. G. (2010). Dynamic three-dimensional culture methods enhance mesenchymal stem cell properties and increase therapeutic potential. *Tissue Eng. Part C Methods* 16 735–749. 10.1089/ten.TEC.2009.0432 19811095

[B38] Guilak F. Estes B. T. Diekman B. O. Moutos F. T. Gimble J. M. (2010). 2010 nicolas andry award: multipotent adult stem cells from adipose tissue for musculoskeletal tissue engineering. *Clin. Orthopaedics Relat. Res.* 468 2530–2540. 10.1007/s11999-010-1410-9 20625952 PMC2919887

[B39] Gutzweiler L. Kartmann S. Troendle K. Benning L. Finkenzeller G. Zengerle R. (2017). Large scale production and controlled deposition of single HUVEC spheroids for bioprinting applications. *Biofabrication* 9:025027. 10.1088/1758-5090/aa7218 28488594

[B40] Hass R. Kasper C. Böhm S. Jacobs R. (2011). Different populations and sources of human mesenchymal stem cells (MSC): a comparison of adult and neonatal tissue-derived MSC. *Cell Commun. Signal.* 9:12. 10.1186/1478-811X-9-12 21569606 PMC3117820

[B41] Hsu S.-H. Hsieh P.-S. (2015). Self-assembled adult adipose-derived stem cell spheroids combined with biomaterials promote wound healing in a rat skin repair model. *Wound Repair Regen.* 23 57–64. 10.1111/wrr.12239 25421559

[B42] Hsu S. H. Huang G. S. (2013). Substrate-dependent Wnt signaling in MSC differentiation within biomaterial-derived 3D spheroids. *Biomaterials* 34 4725–4738. 10.1016/j.biomaterials.2013.03.031 23562051

[B43] Huang G. S. Dai L. G. Yen B. L. Hsu S. H. (2011). Spheroid formation of mesenchymal stem cells on chitosan and chitosan-hyaluronan membranes. *Biomaterials* 32 6929–6945. 10.1016/j.biomaterials.2011.05.092 21762982

[B44] Ip B. C. Cui F. Tripathi A. Morgan J. R. (2016). The bio-gripper: a fluid-driven micro-manipulator of living tissue constructs for additive bio-manufacturing. *Biofabrication* 8:025015. 10.1088/1758-5090/8/2/025015 27221320

[B45] Jakab K. Norotte C. Damon B. Marga F. Neagu A. Besch-Williford C. L. (2008). Tissue engineering by self-assembly of cells printed into topologically defined structures. *Tissue Eng. Part A* 14 413–421. 10.1089/tea.2007.0173 18333793

[B46] Jones E. Crawford A. English A. Henshaw K. Mundy J. Corscadden D. (2008). Synovial fluid mesenchymal stem cells in health and early osteoarthritis: detection and functional evaluation at the single-cell level. *Arthritis Rheumatism* 58 1731–1740. 18512779 10.1002/art.23485

[B47] Jung S. Sen A. Rosenberg L. Behie L. A. (2010). Identification of growth and attachment factors for the serum-free isolation and expansion of human mesenchymal stromal cells. *Cytotherapy* 12 637–657. 20608762 10.3109/14653249.2010.495113

[B48] Karnieli O. Friedner O. M. Allickson J. G. Zhang N. Jung S. Fiorentini D. (2017). A consensus introduction to serum replacements and serum-free media for cellular therapies. *Cytotherapy* 19 155–169. 10.1016/j.jcyt.2016.11.011 28017599

[B49] Koç O. N. Day J. Nieder M. Gerson S. L. Lazarus H. M. Krivit W. (2002). Allogeneic mesenchymal stem cell infusion for treatment of metachromatic leukodystrophy (MLD) and Hurler syndrome (MPS-IH). *Bone Marrow Transplant* 30 215–222. 10.1038/sj.bmt.1703650 12203137

[B50] Kolettis T. M. Bagli E. Barka E. Kouroupis D. Kontonika M. Vilaeti A. D. (2018). Medium-term electrophysiologic effects of a cellularized scaffold implanted in rats after myocardial infarction. *Cureus* 10:e2959. 10.7759/cureus.2959 30214847 PMC6132679

[B51] Kouroupis D. Bowles A. C. Best T. M. Kaplan L. D. Correa D. (2020a). CD10/Neprilysin enrichment in infrapatellar fat pad–derived mesenchymal stem cells under regulatory-compliant conditions: implications for efficient synovitis and fat pad fibrosis reversal. *Am. J. Sports Med.* 48 2013–2027. 10.1177/0363546520917699 32427493

[B52] Kouroupis D. Bowles A. C. Greif D. N. Leñero C. Best T. M. Kaplan L. D. (2020b). Regulatory-compliant conditions during cell product manufacturing enhance in vitro immunomodulatory properties of infrapatellar fat pad-derived mesenchymal stem/stromal cells. *Cytotherapy* 22 677–689. 10.1016/j.jcyt.2020.06.007 32723596

[B53] Kouroupis D. Sanjurjo-Rodriguez C. Jones E. Correa D. (2018). Mesenchymal stem cell functionalization for enhanced therapeutic applications. *Tissue Eng. Part B* 25 55–77. 10.1089/ten.teb.2018.0118 30165783

[B54] Kouroupis D. Wang X. N. El-Sherbiny Y. McGonagle D. Jones E. (2017). “The safety of non-expanded multipotential stromal cell therapies,” in *Safety, Ethics and Regulations*, eds Pham P. V. Rosemann A. (Cham: Springer International Publishing), 91–118.

[B55] Kouroupis D. Willman M. A. Best T. M. Kaplan L. D. Correa D. (2021). Infrapatellar fat pad-derived mesenchymal stem cell-based spheroids enhance their therapeutic efficacy to reverse synovitis and fat pad fibrosis. *Stem Cell Res. Ther.* 12:44. 10.1186/s13287-020-02107-6 PMC7792122 33413649

[B56] Krampera M. Galipeau J. Shi Y. Tarte K. Sensebe L. (2013). Immunological characterization of multipotent mesenchymal stromal cells—The international society for cellular therapy (ISCT) working proposal. *Cytotherapy* 15 1054–1061. 10.1016/j.jcyt.2013.02.010 23602578

[B57] Le Blanc K. Frassoni F. Ball L. Locatelli F. Roelofs H. Lewis I. (2008). Mesenchymal stem cells for treatment of steroid-resistant, severe, acute graft-versus-host disease: a phase II study. *Lancet* 371 1579–1586. 18468541 10.1016/S0140-6736(08)60690-X

[B58] Lee E. J. Park S. J. Kang S. K. Kim G.-H. Kang H.-J. Lee S.-W. (2012). Spherical bullet formation via e-cadherin promotes therapeutic potency of mesenchymal stem cells derived from human umbilical cord blood for myocardial infarction. *Mol. Ther.* 20 1424–1433. 10.1038/mt.2012.58 22453767 PMC3392985

[B59] Lee J. H. Han Y.-S. Lee S. H. (2016). Long-duration three-dimensional spheroid culture promotes angiogenic activities of adipose-derived mesenchymal stem cells. *Biomol. Ther.* 24 260–267. 10.4062/biomolther.2015.146 26869524 PMC4859789

[B60] Lewis E. E. L. Wheadon H. Lewis N. Yang J. Mullin M. Hursthouse A. (2016). A quiescent, regeneration-responsive tissue engineered mesenchymal stem cell bone marrow niche model via magnetic levitation. *ACS Nano* 10 8346–8354. 10.1021/acsnano.6b02841 27602872

[B61] Li Y. Guo G. Li L. Chen F. Bao J. Shi Y.-J. (2015). Three-dimensional spheroid culture of human umbilical cord mesenchymal stem cells promotes cell yield and stemness maintenance. *Cell Tissue Res.* 360 297–307. 10.1007/s00441-014-2055-x 25749992

[B62] Liu B.-H. Yeh H.-Y. Lin Y.-C. Wang M.-H. Chen D. C. Lee B.-H. (2013). Spheroid formation and enhanced cardiomyogenic potential of adipose-derived stem cells grown on chitosan. *BioResearch Open Access* 2 28–39. 10.1089/biores.2012.0285 23514754 PMC3569958

[B63] Liu Z. C. Chang T. M. S. (2006). Transdifferentiation of bioencapsulated bone marrow cells into hepatocyte-like cells in the 90% hepatectomized rat model. *Liver Transplantation* 12 566–572. 10.1002/lt.20635 16496278

[B64] Liu Z. C. Chang T. M. S. (2009). Preliminary study on intrasplenic implantation of artificial cell bioencapsulated stem cells to increase the survival of 90% hepatectomized rats. *Artif. Cells Blood Subst. Biotechnol.* 37 53–55. 10.1080/10731190802663975 19132579 PMC3518483

[B65] Liu Z. C. Chang T. M. S. (2012). Intrasplenic transplantation of bioencapsulated mesenchymal stem cells improves the recovery rates of 90% partial hepatectomized rats. *Stem Cells Int.* 2012:697094. 10.1155/2012/697094 23251190 PMC3515999

[B66] Lou Y. Guo D. Zhang H. Song L. (2016). Effectiveness of mesenchymal stems cells cultured by hanging drop vs. conventional culturing on the repair of hypoxic-ischemic-damaged mouse brains, measured by stemness gene expression. *Open Life Sci.* 11 519–523.

[B67] Ma D. Zhong C. Yao H. Liu Y. Chen F. Li J. (2011). Engineering injectable bone using bone marrow stromal cell aggregates. *Stem Cells Dev.* 20 989–999. 10.1089/scd.2010.0348 21091305

[B68] Mackay A. M. Beck S. C. Murphy J. M. Barry F. P. Chichester C. O. Pittenger M. F. (1998). Chondrogenic differentiation of cultured human mesenchymal stem cells from marrow. *Tissue Eng.* 4 415–428. 10.1089/ten.1998.4.415 9916173

[B69] Markou M. Kouroupis D. Badounas F. Katsouras A. Kyrkou A. Fotsis T. (2020). Tissue engineering using vascular organoids from human pluripotent stem cell derived mural cell phenotypes. *Front. Bioeng. Biotechnol.* 8:278. 10.3389/fbioe.2020.00278 32363181 PMC7182037

[B70] Mekhileri N. V. Lim K. S. Brown G. C. J. Mutreja I. Schon B. S. Hooper G. J. (2018). Automated 3D bioassembly of micro-tissues for biofabrication of hybrid tissue engineered constructs. *Biofabrication* 10:024103. 10.1088/1758-5090/aa9ef1 29199637

[B71] Menard C. Pacelli L. Bassi G. Dulong J. Bifari F. Bezier I. (2013). Clinical-grade mesenchymal stromal cells produced under various good manufacturing practice processes differ in their immunomodulatory properties: standardization of immune quality controls. *Stem Cells Dev.* 22 1789–1801. 10.1089/scd.2012.0594 23339531 PMC3668498

[B72] Mendicino M. Bailey Alexander M. Wonnacott K. Puri R. K. Bauer (2014). MSC-based product characterization for clinical trials: an FDA perspective. *Cell Stem Cell* 14 141–145. 10.1016/j.stem.2014.01.013 24506881

[B73] Meng R. Xu H.-Y. Di S.-M. Shi D.-Y. Qian A.-R. Wang J.-F. (2011). Human mesenchymal stem cells are sensitive to abnormal gravity and exhibit classic apoptotic features. *Acta Bioch. Biophys. Sin.* 43 133–142. 10.1093/abbs/gmq121 21266543

[B74] Messina A. Morelli S. Forgacs G. Barbieri G. Drioli E. De Bartolo L. (2017). Self-assembly of tissue spheroids on polymeric membranes. *J. Tissue Eng. Regen. Med.* 11 2090–2103. 10.1002/term.2105 26549598

[B75] Meyers V. E. Zayzafoon M. Douglas J. T. McDonald J. M. (2005). RhoA and cytoskeletal disruption mediate reduced osteoblastogenesis and enhanced adipogenesis of human mesenchymal stem cells in modeled microgravity. *J. Bone Min. Res.* 20 1858–1866. 10.1359/JBMR.050611 16160744 PMC1351020

[B76] Meyers V. E. Zayzafoon M. Gonda S. R. Gathings W. E. McDonald J. M. (2004). Modeled microgravity disrupts collagen I/integrin signaling during osteoblastic differentiation of human mesenchymal stem cells. *J. Cell. Biochem.* 93 697–707. 10.1002/jcb.20229 15660414

[B77] Mironov V. Visconti R. P. Kasyanov V. Forgacs G. Drake C. J. Markwald R. R. (2009). Organ printing: tissue spheroids as building blocks. *Biomaterials* 30 2164–2174. 10.1016/j.biomaterials.2008.12.084 19176247 PMC3773699

[B78] Moldovan N. I. Hibino N. Nakayama K. (2017). Principles of the kenzan method for robotic cell spheroid-based three-dimensional bioprinting. *Tissue Eng. Part B Rev.* 23 237–244. 10.1089/ten.TEB.2016.0322 27917703

[B79] Moretti P. Hatlapatka T. Marten D. Lavrentieva A. Majore I. Hass R. (2010). Mesenchymal stromal cells derived from human umbilical cord tissues: primitive cells with potential for clinical and tissue engineering applications. *Adv. Biochem. Eng. Biotechnol.* 123 29–54. 10.1007/10_2009_15 20012739

[B80] Mueller-Klieser W. (1984). Method for the determination of oxygen consumption rates and diffusion coefficients in multicellular spheroids. *Biophys. J.* 46 343–348. 10.1016/s0006-3495(84)84030-8 6487734 PMC1434957

[B81] Murphy K. C. Fang S. Y. Leach J. K. (2014). Human mesenchymal stem cell spheroids in fibrin hydrogels exhibit improved cell survival and potential for bone healing. *Cell Tissue Res.* 357 91–99. 10.1007/s00441-014-1830-z 24781147 PMC4077909

[B82] Murphy K. C. Hung B. P. Browne-Bourne S. Zhou D. Yeung J. Genetos D. C. (2017). Measurement of oxygen tension within mesenchymal stem cell spheroids. *J. R. Soc. Interface* 14:20160851. 10.1098/rsif.2016.0851 28179546 PMC5332570

[B83] Nikolova M. P. Chavali M. S. (2019). Recent advances in biomaterials for 3D scaffolds: a review. *Bioact. Mater.* 4 271–292. 10.1016/j.bioactmat.2019.10.005 31709311 PMC6829098

[B84] Petrenko Y. Syková E. Kubinová Š (2017). The therapeutic potential of three-dimensional multipotent mesenchymal stromal cell spheroids. *Stem Cell Res. Ther.* 8:94. 10.1186/s13287-017-0558-6 28446248 PMC5406927

[B85] Pittenger M. F. Discher D. E. Péault B. M. Phinney D. G. Hare J. M. Caplan A. I. (2019). Mesenchymal stem cell perspective: cell biology to clinical progress. *npj Regen. Med.* 4:22. 10.1038/s41536-019-0083-6 31815001 PMC6889290

[B86] Potapova I. A. Brink P. R. Cohen I. S. Doronin S. V. (2008). Culturing of human mesenchymal stem cells as three-dimensional aggregates induces functional expression of CXCR4 that regulates adhesion to endothelial cells. *J. Biol. Chem.* 283 13100–13107. 10.1074/jbc.M800184200 18334485 PMC2442325

[B87] Potapova I. A. Gaudette G. R. Brink P. R. Robinson R. B. Rosen M. R. Cohen I. S. (2007). Mesenchymal stem cells support migration, extracellular matrix invasion, proliferation, and survival of endothelial cells in vitro. *Stem Cells* 25 1761–1768. 17395769 10.1634/stemcells.2007-0022

[B88] Redondo-Castro E. Cunningham C. J. Miller J. Brown H. Allan S. M. Pinteaux E. (2018a). Changes in the secretome of tri-dimensional spheroid-cultured human mesenchymal stem cells in vitro by interleukin-1 priming. *Stem Cell Res. Ther.* 9:11. 10.1186/s13287-017-0753-5 29343288 PMC5773162

[B89] Redondo-Castro E. Cunningham C. J. Miller J. Cain S. A. Allan S. M. Pinteaux E. (2018b). Generation of Human mesenchymal stem cell 3D spheroids using low-binding plates. *Bio Protoc.* 8:e2968. 10.21769/BioProtoc.2968 30294619 PMC6173304

[B90] Ringden O. Uzunel M. Rasmusson I. Remberger M. Sundberg B. Lonnies H. (2006). Mesenchymal stem cells for treatment of therapy-resistant graft-versus-host disease. *Transplantation* 81 1390–1397. 16732175 10.1097/01.tp.0000214462.63943.14

[B91] Sart S. Tsai A. C. Li Y. Ma T. (2014). Three-dimensional aggregates of mesenchymal stem cells: cellular mechanisms, biological properties, and applications. *Tissue Eng. Part B Rev.* 20 365–380. 10.1089/ten.TEB.2013.0537 24168395 PMC4185975

[B92] Seda Tığlı R. Karakeçili A. Gümüşderelioğlu M. (2007). In vitro characterization of chitosan scaffolds: influence of composition and deacetylation degree. *J. Mater. Sci.* 18 1665–1674. 10.1007/s10856-007-3066-x 17483879

[B93] Sheyn D. Pelled G. Netanely D. Domany E. Gazit D. (2010). The effect of simulated microgravity on human mesenchymal stem cells cultured in an osteogenic differentiation system: a bioinformatics study. *Tissue Eng Part A* 16 3403–3412. 10.1089/ten.tea.2009.0834 20807102 PMC2971652

[B94] Singer N. G. Caplan A. I. (2011). Mesenchymal stem cells: mechanisms of inflammation. *Annu. Rev. Pathol.* 6 457–478. 10.1146/annurev-pathol-011110-130230 21073342

[B95] Sorrell J. M. Baber M. A. Caplan A. I. (2009). Influence of adult mesenchymal stem cells on in vitro vascular formation. *Tissue Eng Part A* 15 1751–1761. 10.1089/ten.tea.2008.0254 19196139 PMC2792097

[B96] Suenaga H. Furukawa K. S. Suzuki Y. Takato T. Ushida T. (2015). Bone regeneration in calvarial defects in a rat model by implantation of human bone marrow-derived mesenchymal stromal cell spheroids. *J. Mater. Sci. Mater. Med.* 26:254. 10.1007/s10856-015-5591-3 26449444 PMC4598349

[B97] Suzuki S. Muneta T. Tsuji K. Ichinose S. Makino H. Umezawa A. (2012). Properties and usefulness of aggregates of synovial mesenchymal stem cells as a source for cartilage regeneration. *Arthritis Res. Ther.* 14:R136. 10.1186/ar3869 PMC3446519 22676383

[B98] Theisen C. S. Wahl J. K. III Johnson K. R. Wheelock M. J. (2007). NHERF links the N-cadherin/catenin complex to the platelet-derived growth factor receptor to modulate the actin cytoskeleton and regulate cell motility. *Mol. Biol. Cell* 18 1220–1232. 10.1091/mbc.e06-10-0960 17229887 PMC1838972

[B99] Toma C. Pittenger Mark F. Cahill Kevin S. Byrne Barry J. Kessler Paul D. (2002). Human mesenchymal stem cells differentiate to a cardiomyocyte phenotype in the adult murine heart. *Circulation* 105 93–98. 10.1161/hc0102.101442 11772882

[B100] Toma C. Wagner W. R. Bowry S. Schwartz A. Villanueva F. (2009). Fate of culture-expanded mesenchymal stem cells in the microvasculature: in vivo observations of cell kinetics. *Circ. Res.* 104 398–402. 10.1161/CIRCRESAHA.108.187724 19096027 PMC3700384

[B101] Uccelli A. Rosbo N. K. (2015). The immunomodulatory function of mesenchymal stem cells: mode of action and pathways. *Ann. N. Y. Acad. Sci.* 1351 114–126. 10.1111/nyas.12815 26152292

[B102] Vorwald C. E. Ho S. S. Whitehead J. Leach J. K. (2018). High-throughput formation of mesenchymal stem cell spheroids and entrapment in alginate hydrogels. *Methods Mol. Biol.* 1758 139–149. 10.1007/978-1-4939-7741-3_11 29679328 PMC6764926

[B103] Wang C.-C. Chen C.-H. Hwang S.-M. Lin W.-W. Huang C.-H. Lee W.-Y. (2009). Spherically symmetric mesenchymal stromal cell bodies inherent with endogenous extracellular matrices for cellular cardiomyoplasty. *Stem Cells* 27 724–732. 10.1634/stemcells.2008-0944 19259939

[B104] Wang W. Itaka K. Ohba S. Nishiyama N. Chung U.-I. Yamasaki Y. (2009). 3D spheroid culture system on micropatterned substrates for improved differentiation efficiency of multipotent mesenchymal stem cells. *Biomaterials* 30 2705–2715. 10.1016/j.biomaterials.2009.01.030 19215979

[B105] Waterman R. S. Tomchuck S. L. Henkle S. L. Betancourt A. M. (2010). A new mesenchymal stem cell (MSC) paradigm: polarization into a pro-inflammatory MSC1 or an immunosuppressive MSC2 phenotype. *PLoS One* 5:e10088. 10.1371/journal.pone.0010088 20436665 PMC2859930

[B106] Weiss M. Troyer D. (2006). Stem cells in the umbilical cord. *Stem Cell Rev. Rep.* 2 155–162. 10.1007/s12015-006-0022-y PMC3753204 17237554

[B107] Xu L. Meng F. Ni M. Lee Y. Li G. (2013). N-cadherin regulates osteogenesis and migration of bone marrow-derived mesenchymal stem cells. *Mol. Biol. Rep.* 40 2533–2539. 10.1007/s11033-012-2334-0 23187741

[B108] Xu Y. Shi T. Xu A. Zhang L. (2016). 3D spheroid culture enhances survival and therapeutic capacities of MSCs injected into ischemic kidney. *J. Cell. Mol.Med.* 20 1203–1213. 10.1111/jcmm.12651 26914637 PMC4929304

[B109] Yanagihara K. Uchida S. Ohba S. Kataoka K. Itaka K. (2018). Treatment of bone defects by transplantation of genetically modified mesenchymal stem cell spheroids. *Mol. Ther. Methods Clin. Dev.* 9 358–366. 10.1016/j.omtm.2018.04.006 30038939 PMC6054700

[B110] Yang C. M. Huang Y. J. Hsu S. H. (2015). Enhanced autophagy of adipose-derived stem cells grown on chitosan substrates. *Biores. Open Access* 4 89–96. 10.1089/biores.2014.0032 26309785 PMC4497627

[B111] Yeh H.-Y. Liu B.-H. Hsu S.-H. (2012). The calcium-dependent regulation of spheroid formation and cardiomyogenic differentiation for MSCs on chitosan membranes. *Biomaterials* 33 8943–8954. 10.1016/j.biomaterials.2012.08.069 22985995

[B112] Yeh H.-Y. Liu B.-H. Sieber M. Hsu S.-H. (2014). Substrate-dependent gene regulation of self-assembled human MSC spheroids on chitosan membranes. *BMC Genomics* 15:10. 10.1186/1471-2164-15-10 24387160 PMC4046657

[B113] Ylöstalo J. H. Bartosh T. J. Coble K. Prockop D. J. (2012). Human mesenchymal stem/stromal cells (hMSCs) cultured as spheroids are self-activated to produce prostaglandin E2 (PGE2) that directs stimulated macrophages into an anti-inflammatory phenotype. *Stem Cells* 30 2283–2296. 10.1002/stem.1191 22865689 PMC3448872

[B114] Ylostalo J. H. Bartosh T. J. Tiblow A. Prockop D. J. (2014). Unique characteristics of human mesenchymal stromal/progenitor cells pre-activated in 3-dimensional cultures under different conditions. *Cytotherapy* 16 1486–1500. 10.1016/j.jcyt.2014.07.010 25231893 PMC4190045

[B115] Ylostalo J. H. Bazhanov N. Mohammadipoor A. Bartosh T. J. (2017). Production and administration of therapeutic mesenchymal stem/stromal cell (MSC) spheroids primed in 3-D cultures under xeno-free conditions. *J. Vis. Exp.* 121 55126. 10.3791/55126 28362380 PMC5409342

[B116] Yu B. Yu D. Cao L. Zhao X. Long T. Liu G. (2011). Simulated microgravity using a rotary cell culture system promotes chondrogenesis of human adipose-derived mesenchymal stem cells via the p38 MAPK pathway. *Biochem. Biophys. Res. Commun.* 414 412–418. 10.1016/j.bbrc.2011.09.103 21971552

[B117] Zhang K. Yan S. Li G. Cui L. Yin J. (2015). In-situ birth of MSCs multicellular spheroids in poly(l-glutamic acid)/chitosan scaffold for hyaline-like cartilage regeneration. *Biomaterials* 71 24–34. 10.1016/j.biomaterials.2015.08.037 26318814

[B118] Zhang Q. Nguyen A. L. Shi S. Hill C. Wilder-Smith P. Krasieva T. B. (2012). Three-dimensional spheroid culture of human gingiva-derived mesenchymal stem cells enhances mitigation of chemotherapy-induced oral mucositis. *Stem Cells Dev.* 21 937–947. 10.1089/scd.2011.0252 21689066 PMC3315752

[B119] Zimmermann J. A. McDevitt T. C. (2014). Pre-conditioning mesenchymal stromal cell spheroids for immunomodulatory paracrine factor secretion. *Cytotherapy* 16 331–345. 10.1016/j.jcyt.2013.09.004 24219905

[B120] Zuk P. A. Zhu M. Ashjian P. De Ugarte D. A. Huang J. I. Mizuno H. (2002). Human adipose tissue is a source of multipotent stem cells. *Mol. Biol. Cell* 13 4279–4295. 12475952 10.1091/mbc.E02-02-0105 PMC138633

